# High medical costs and resource shortages constrain rural happiness more than physical access in rural China

**DOI:** 10.3389/fpubh.2026.1822849

**Published:** 2026-05-15

**Authors:** Yuchen He, Shan Jin, Jing Chen

**Affiliations:** 1 Institute of Agricultural Economics and Development, Chinese Academy of Agricultural Sciences, Beijing, China; 2 Department of Land and Property Management, Royal Agricultural University, Cirencester, United Kingdom; 3 Faculty of Business and Law, University of Portsmouth, Portsmouth, United Kingdom

**Keywords:** accessibility, happiness, healthcare service, medical, rural residents

## Abstract

**Introduction:**

Access to healthcare services is closely associated with people’s subjective well-being (SWB). Although a growing body of research has examined this relationship in rural contexts, variations across different rural regions in China remain underexplored.

**Methods:**

This study investigates the association between multidimensional healthcare accessibility—specifically affordability, availability, and geographic accessibility—and the SWB of 589 rural residents from two distinct counties representing economically developed (Kunshan, Jiangsu) and less-developed (Yudu, Jiangxi) regions.

**Results:**

Affordability and resource availability are significantly associated with rural residents’ happiness, whereas geographic distance is no longer a primary constraint. Significant regional heterogeneity exists in mediation pathways: in the less-developed region, availability acts as a psychological safety net linked to reduced anxiety; in the developed region, affordability constraints are linked to reduced happiness through physical health capital depreciation.

**Discussion:**

Public health governance should transition from universal spatial expansion toward precision-driven interventions focusing on medical expenditure and service quality to better support the happiness of diverse rural populations.

## 1 Introduction

Subjective well-being (SWB, hereafter referred to as happiness), which is often conceptualized as happiness or life satisfaction, has emerged as a critical indicator of social progress, transcending traditional economic metrics such as GDP. Among the numerous factors associated with happiness, health status is consistently identified as one of the most robust correlates (1, 2). According to the capability approach proposed by Sen, health is not merely a biological state but a fundamental freedom that enables individuals to pursue the lives they want (3). In contemporary society, an individual’s health status is closely linked to the healthcare services and relevant education they receive. Therefore, ensuring that different groups of people have access to the healthcare services they need is widely recognized as essential for supporting residents’ happiness. Specifically, for vulnerable populations in developing countries, the friction of distance, the shortage of essential medicines at local clinics, and the catastrophic risk of unaffordable medical expenditure are all barriers to accessing healthcare services, which may create a “health poverty trap” (4). This trap implies a vicious cycle where poor health depletes economic assets, and economic poverty acts as a barrier to healthcare, subsequently eroding both physical and mental happiness. In Vietnam, for instance, during its economic development, reforms to the public health system left poor households highly vulnerable to catastrophic health expenditures, potentially consuming up to 21.9% of their total income (5). Therefore, understanding residents’ needs and the association between healthcare accessibility and happiness is crucial for formulating effective public health policies worldwide.

As the world’s largest developing economy, China presents a paradigmatic case for examining these dynamics. Over the past two decades, China has implemented massive healthcare reforms, most notably the New Rural Cooperative Medical Scheme, which has achieved near-universal coverage for the rural population (6). The New Rural Cooperative Medical Scheme is a massive, government-driven system set up to extend health insurance to the rural population. Its key actions center on heavy public financing, voluntary household enrollment, and a focus on catastrophic inpatient coverage. These efforts have significantly reduced the financial burden of illness for rural residents, despite a significant gap in living standards between different regions, especially between urban and rural areas (7). Data from the World Happiness Report indicate a continuous decline in China’s happiness index ranking from 2016 to 2020 (8). Notably, the growth rate of happiness scores for rural residents has lagged 1.2 percentage points behind their urban counterparts, with the “health poverty trap” considered a key impediment to closing this gap (9). This challenge has been exacerbated by China’s rapidly aging population and the different institutional arrangements in urban and rural society (10). Rural areas are aging faster than urban centers, leading to a surging demand for chronic disease management and mental health support. Yet, the supply side remains imbalanced: high-quality medical resources—such as tertiary hospitals and senior specialists—are heavily concentrated in urban areas, leaving rural clinics under-resourced. This structural inequality means that for many rural residents, medical services may be financially subsidized but practically unavailable due to a lack of equipment or qualified doctors in their vicinity (11). Recent studies highlight that this inequality of opportunity in healthcare is a major source of dissatisfaction among rural residents, potentially undermining the social stability and happiness gains expected from economic growth (12).

In recent years, a substantial body of literature has examined the urban–rural health gap. However, the literature often aggregates data across vast geographical areas or focuses solely on impoverished regions in Western China (13). This approach may obscure the significant intra-rural heterogeneity that has emerged after decades of uneven regional development, which may result in generalized policies failing to address the health inequality across diverse rural populations (14). For example, in economically developed rural areas, residents may benefit from higher income levels and better transport infrastructure to access urban medical resources; conversely, in underdeveloped regions, the lack of basic medical equipment (availability) remains the primary bottleneck associated with lower happiness. To address this knowledge gap, the current study conducts a comparative analysis of two distinct rural counties in China: Kunshan in Jiangsu Province and Yudu in Jiangxi Province. Kunshan, located in the Yangtze River Delta, represents one of the most economically developed rural areas in China, with high levels of urbanization and industrialization. Yudu, located in central China, represents a typical medical-resource-deprived region despite recent economic development. Our study proposes and tests a comprehensive theoretical framework to analyze the associations between three dimensions of healthcare accessibility—affordability, geographic accessibility, and availability—and rural residents’ happiness. Given that health is theoretically conceptualized as a key link connecting medical services and happiness, we incorporate both Physical Health and Mental Health as mediating variables to explore the underlying correlational pathways.

## 2 Literature review and theoretical background

### 2.1 Theoretical framework

This study is grounded in two complementary theoretical frameworks: Amartya Sen’s capability approach and Andersen’s behavioral model of health services use. Sen suggests that health is a fundamental capability, and the expansion of such capabilities represents the real freedom individuals have to achieve the lives they value (3). A lack of access to healthcare is not merely a service deprivation but a capability failure that is directly associated with lower happiness. Andersen’s model distinguishes between potential access (enabling resources like insurance and facility presence) and realized access (actual utilization) (15). In the context of rural China, this framework can help explain why high insurance coverage (affordability) does not always correspond to higher happiness if availability (e.g., of equipment or medicines) is lacking (4).

### 2.2 Healthcare accessibility and subjective well-being

Healthcare accessibility is a multidimensional construct. Drawing on the seminal framework by Penchansky and Thomas, this study focuses on three dimensions critical to rural residents: affordability, geographic accessibility, and availability (16). Affordability is directly linked to residents’ economic security. High out-of-pocket expenses not only correlated with the crowding out of household consumption in other domains but also linked to severe anxiety regarding poverty caused by illness. This psychological distress has proved to be a significant factor in reducing happiness (17). Geographic accessibility reflects the time costs of, and physical barriers to, obtaining services. Proximity to medical institutions influences medical-seeking behavior; a convenient geographic location provides a sense of security, alleviating residents’ concerns about delayed treatment for sudden illnesses (18–20). Availability refers to the adequacy of medical resources, such as pharmaceuticals, equipment, and medical personnel. For rural residents, the prevalent shortage of medicine and doctors in basic, locally run health institutions often exacerbates a sense of helplessness and mistrust toward the medical system (19, 21). Existing studies suggest that improvements in any of these dimensions can significantly enhance life satisfaction by alleviating financial pressure, increasing perceived security, and improving service satisfaction (10, 22). As such, the following hypothesis has been proposed:

*H1*: The three dimensions of rural healthcare accessibility are significantly and positively associated with the happiness of rural residents.

### 2.3 Regional heterogeneity

Although healthcare accessibility is universally important, its marginal effect on happiness depends on the level of economic development in a particular region; thus, the varying statistical relationships between accessibility and happiness across different regions warrant attention in policymaking (23). Rural China is not a homogeneous entity; regions at different levels of development face distinct medical constraints (24, 25). In economically underdeveloped agricultural areas (such as Yudu County, selected for the current study), medical infrastructure tends to be relatively weak. The shortage of pharmaceuticals and basic diagnostic equipment (i.e., insufficient availability) is often the primary impediment to the fulfillment of residents’ health needs (11). Consequently, in such regions, even marginal improvements in medical resource supply may be correlated with a significant leap in residents’ happiness by addressing the most critical gaps in basic care. Conversely, in economically developed rural areas (such as Kunshan in the current study), basic medical facilities are very likely to be approaching saturation. As residents’ basic medical needs are well-met, their sensitivity to resource availability may diminish, shifting their focus toward service quality and other dimensions (26, 27). As such, the current study argues that resource constraints render underdeveloped regions more sensitive to changes in availability than their more developed counterparts. The following hypothesis has thus been proposed:

*H2*: The association between healthcare accessibility and happiness varies across regions with different levels of economic development. Specifically, the positive statistical relationship between medical resource availability and happiness is stronger in underdeveloped rural areas compared to developed rural areas.

### 2.4 Mediating role

The association between healthcare accessibility and happiness does not exist in a vacuum; rather, it may be statistically linked through the maintenance and improvement of residents’ health stock. Drawing on Grossman’s Health Capital Model, medical services are viewed as inputs to produce health capital (28, 29). It is the stock of health, rather than the medical service itself, that is most directly correlated with an individual’s utility and happiness. On the one hand, good healthcare accessibility (e.g., timely diagnosis and affordable treatment) is closely associated with better control over the progression of chronic diseases and maintain physical functioning. This safeguards residents’ labor income capacity and ability to perform daily living activities, which is particularly critical for rural residents reliant on manual labor and constitutes the material basis of happiness (30). On the other hand, with the intensifying aging of China’s rural population, the security effect of medical services has become increasingly salient. Convenient and adequate medical resources serve as a social safety net, significantly reducing catastrophic expectations of future health risks and psychological anxiety among the older adults (31, 32). Especially from a comparative regional perspective, residents in developed areas may derive happiness primarily through improvements in physical health (maintenance of labor capacity) and enhanced consumption ability (33, 34). In contrast, residents in regions with lower economic development or older populations may derive happiness more from the maintenance of mental health (reduction of anxiety) (35, 36). Accordingly, the following hypothesis is proposed:

*H3*: Physical health and mental health serve as statistical mediators in the association between healthcare accessibility and the happiness of rural residents.

## 3 Materials and methods

### 3.1 Survey design

The questionnaire design was informed by previous research into healthcare accessibility, health capital, and happiness. Following the established methodologies in large-scale social surveys such as the World Values Survey and previous influential studies, happiness was measured using a single-item global assessment (37, 38). Specifically, respondents were asked: “Overall, do you feel that your life is happy right now?” Responses were recorded on a 5-point Likert scale ranging from 1 (“Very unhappy”) to 5 (“Very happy”).

To systematically evaluate the multidimensional health status of rural residents, this study employed the 12-Item Short Form Health Survey (SF-12), a widely validated instrument for assessing health-related quality of life (HRQoL) (39). The SF-12 is a shortened version of the SF-36 and was selected for this study to minimize respondent burden among the rural population while maintaining high reliability and validity (40). It covers two distinct composite scores: the Physical Component Summary (PCS) and the Mental Component Summary (MCS). Previous studies have demonstrated the strong psychometric properties of the SF-12 in Chinese populations (41).

In terms of healthcare accessibility, this variable was measured across three specific dimensions: affordability, geographic accessibility, and availability (16). Following the standard methodology in health equity research (42), affordability was operationalized as the ratio of annual out-of-pocket medical expenditure to total annual household income. A higher ratio signifies lower affordability and higher financial risk, which has been identified as a key correlate negatively associated with happiness among rural Chinese residents (11). Geographic accessibility was operationalized as the one-way travel time (in minutes) required for residents to travel from their homes to the nearest medical institution using their usual mode of transportation (43), a metric widely acknowledged in health research. Availability refers to the adequacy of the supply of medical resources relative to patient needs (20). In the context of rural China, the shortage of essential medicines and diagnostic equipment has been identified as a critical structural quality bottleneck (12). Therefore, this study measured availability by asking respondents to rate the frequency of encountering shortages of essential medicines and equipment at their local health institutions on a 4-point scale (1 = “Always Unavailable” to 4 = “Available & Sufficient”). Scores were coded, with higher values indicating greater resource availability (44).

The final questionnaire also covered basic personal and family demographic information, including age, gender, education level, marital status, labor status, annual household income, and so on. The definitions of all variables can be found in Table 1.

**Table 1:** Description of variable design and descriptive statistics.

Variables	Measures
Dependent variable	
SWB	1 = Very unhappy, 2 = Unhappy, 3 = Neutral, 4 = Happy, 5 = Very happy
Independent variable	
Affordability	Annual health payment/Annual income ratio
Geographical accessibility	Time to medical facility (in minutes)
Availability	1 = Always unavailable, 2 = Sometimes unavailable, 3 = Basically satisfied, 4 = Available and sufficient
Control variables	
Age	Age (years)
Gender	1 = Male; 0 = Female
Education	1 = Primary school and below, 2 = Junior high school, 3 = High school/Vocational, 4 = College/University, 5 = Graduate
Income	Annual personal income of residents: 1 = 0–1, 2 = 1–3, 3 = 3–5, 4 = 5–8, 5 = 8–10, 6 = 10–12, 7 = 12 + (in 10,000 yuan)
Marital status	1 = Married; 0 = Unmarried
Occupation	1 = Farmer, 2 = Self-employed, 3 = Employee/Worker, 4 = Unemployed and others
Health consciousness	Health awareness scale score: 0–100, with higher scores indicating better health awareness
Satisfaction	Residents’ evaluation of village public service conditions: 1 = Very poor, 2 = Poor, 3 = Average, 4 = Good, 5 = Very Good
Mechanism variables	
Physical component summary	A continuous variable ranging from 0 to 100, with higher scores indicating better health status.
Mental component summary	A continuous variable ranging from 0 to 100, with higher scores indicating better health status.

This study aimed to use survey data collected from rural residents to establish a comprehensive understanding of the correlation between multidimensional accessibility and happiness in a Chinese rural context, while considering the significant regional disparities in economic development. To obtain accurate micro-level data required for this study, relevant experts were first consulted to review and evaluate the initial questionnaire template, after which the initial questionnaire was revised. Subsequently, the author conducted a small-scale pilot survey in Yushan Town, Kunshan City, using the modified questionnaire. Based on the responses collected during the pilot survey, further issues identified in the pilot survey were discussed with experts and scholars, leading to another round of revisions.

### 3.2 Data collection

The formal survey was conducted in 2024, covering 108 administrative villages across 31 townships in Yudu County and Kunshan City. These two locations were strategically selected to maximize regional heterogeneity, creating a representative dichotomy of rural China’s development landscape. Kunshan City, situated in the Yangtze River Delta, serves as a proxy for the economically advanced, highly industrialized, and flat-terrain eastern coastal regions, typically characterized by superior medical resource density and transportation networks. In sharp contrast, Yudu County represents the developing inland regions of central China; it features predominantly mountainous and hilly terrain with a traditional agricultural economy, where residents often face many constraints in healthcare delivery and geographic accessibility. This comparison allows for a robust examination of the association between healthcare accessibility and happiness across different stages of economic development.

To ensure sample representativeness, a stratified random sampling method was employed during fieldwork. The survey targeted rural residents aged 18 and above. A mixed-mode data collection approach was adopted to accommodate varying literacy levels: self-administered questionnaires were provided to respondents with higher education levels, while trained investigators conducted one-on-one in-person interviews with those with limited literacy to ensure comprehension and data quality. A total of 639 questionnaires were initially distributed. After a rigorous data cleaning process to exclude incomplete or logically inconsistent responses, 589 valid questionnaires were retained, yielding a high effective response rate of 92.2%. The final analytical sample consisted of 278 respondents from Kunshan and 311 respondents from Yudu, providing a balanced dataset for comparative analysis. To compare the relative importance of the three dimensions in relation to happiness, this study employed the Wald test to assess whether there were statistically significant differences among the coefficients of each variable.

### 3.3 Empirical model

Since the dependent variable in this paper is an ordered variable of rural residents’ happiness, we chose the ordered Logit model for analysis and constructed the following Equation (1):


$$\text{logit}(P(\mathrm{Hap}\leq j))=\alpha_{j}(\begin{array}{l} \beta \mathrm{Acc}+\gamma_{1}\text{gend}+\gamma_{2}\mathrm{Age}+\gamma_{3}\mathrm{Inc}+\\ \gamma_{4}\mathrm{Edu}+\gamma_{5}\mathrm{Occ}+\gamma_{6}\mathrm{Mar}+\\ \gamma_{7}\mathrm{hea}\_c+\gamma_{8}\text{Satisf} \end{array})$$ (1)

In Equation (1), Hap is the explained variable rural residents’ happiness, Acc is the score of rural medical and healthcare accessibility, and gend, Age, Inc. and other control variables represent gender, age, income and so on. j represents the category of the explained variable Hap (from 1 to 5), and $a_{j}$ is the threshold (intercept) for category j , which increases with j . β is the coefficient of the explanatory variable Acc, while $\gamma_{1}$ , $\gamma_{2}$ to $\gamma_{8}$ are the coefficients of control variables such as gender and age.


$$\begin{array}{l} \text{logit}(P(\mathrm{Hap}\leq j))=\alpha_{j}-(\beta 1\mathrm{Aff}+\beta 2\mathrm{Ava}+\beta 3\mathrm{Gac}+\gamma_{1a}\text{gend}+\\ \gamma_{2a}\mathrm{Age}+\gamma_{3a}\mathrm{Inc}+\gamma_{4a}\mathrm{Edu}+\gamma_{5a}\mathrm{Occ}+\\ \gamma_{6a}\mathrm{Mar}+\gamma_{7a}\mathrm{hea}\_c+\gamma_{8a}\text{Satisf}) \end{array}$$ (2)

In Equation (2), Hap is the explained variable rural residents’ happiness, Aff is affordability, Ava is availability, Gac is geographical accessibility.

Since the mediator variables MCS and PCS are continuous variables, in order to study the statistical pathways linking rural medical and healthcare accessibility with rural residents’ happiness, the following mediation effect model is established:


$$\begin{array}{l} \mathrm{MCS}=\beta_{0i}+\beta 1\text{iAff}+\beta 2\text{iAva}+\beta 3\text{iGac}+\delta_{1i}\text{gend}+\\ \delta_{2i}\mathrm{Age}+\delta_{3i}\mathrm{Inc}+\delta_{4i}\mathrm{Edu}+\delta_{5i}\mathrm{Voc}+\\ \delta_{6i}\mathrm{Mar}+\delta_{7i}\mathrm{hea}\_c+\delta_{8i}\text{Satisf}+\varepsilon_{\mathrm{ij}} \end{array}$$ (3)

$$\begin{array}{l} \mathrm{PCS}=\beta_{0j}+\beta 1\text{jAff}+\beta 2\text{jAva}+\beta 3\text{jGac}+\delta_{1j}\text{gend}+\\ \delta_{2j}\mathrm{Age}+\delta_{3j}\mathrm{Inc}+\delta_{4j}\mathrm{Edu}+\delta_{5j}\mathrm{Voc}+\\ \delta_{6j}\mathrm{Mar}+\delta_{7j}\mathrm{hea}\_c+\delta_{8j}\text{Satisf}+\varepsilon_{\mathrm{ij}} \end{array}$$ (4)

$$\text{logit}(P(\mathrm{Hap}\leq j))=\alpha_{j}-(\begin{array}{l} \beta 1^{\prime }\mathrm{Aff}+\beta 2^{\prime }\mathrm{Ava}+\beta 3^{\prime }\mathrm{Gac}+\beta_{a}\mathrm{MCS}+\\ \beta_{b}\mathrm{PCS}+{\gamma_{1}}^{'}\text{gend}+{\gamma_{2}}^{'}\mathrm{Age}+{\gamma_{3}}^{'}\mathrm{Inc}+\\ {\gamma_{4}}^{'}\mathrm{Edu}+{\gamma_{5}}^{'}\mathrm{Voc}+{\gamma_{6}}^{'}\mathrm{Mar}+\\ {\gamma_{7}}^{'}\mathrm{hea}\_c+{\gamma_{8}}^{'}\text{Satisf} \end{array})$$ (5)

In Equations (3)-(5), PCS and MCS represent two mediating variables: physical health and mental health, respectively. $\beta_{0}$ and $\beta_{0j}$ denote the intercept terms of the respective equations, while $\beta_{1i-3i}\;$ and $\beta_{1i-3j}$ indicate the coefficients capturing the association between accessibility and PCS and MCS. The coefficients from $d_{1i}$ to $d_{9i}\;$ and $d_{1j}$ to $d_{8j}$ represent the estimates of control variables on MCS and PCS, with $e_{\mathrm{ij}}$ representing their random error terms. In Equation (5), $\beta_{a}$ and $\beta_{b}$ reflect the coefficients of mediating variables MCS and PCS on rural residents’ happiness.

### 3.4 Ethics statement

This study was conducted in strict accordance with the ethical principles of the Declaration of Helsinki (2013 revision). This study involved human participants and was conducted using anonymous survey data collected from rural residents. All participants provided informed consent prior to participation. The research did not involve clinical trials, biological sample collection, invasive operations, or any procedures that may cause physical or psychological harm to participants. No personally identifiable information (including name, ID number, detailed address, contact information, etc.) was collected during the survey, and all response data were anonymized during collection, storage, and analysis to fully protect the privacy of respondents. All participants were fully informed of the study purpose, survey content, voluntary participation principle, and the right to withdraw from the survey at any time without any adverse consequences before the investigation. Given that this study is a low-risk anonymous social survey with no ethical risks involved, the institutional review board of the Institute of Agricultural Economics and Development, Chinese Academy of Agricultural Sciences confirmed that this study is exempt from formal ethical review and approval.

## 4 Results

### 4.1 Descriptive statistics

Table 2 presents the socio-demographic characteristics and key variables for the total sample (N = 589). The sample is predominantly male (62.48%) and married (89.81%), with a relatively balanced age structure, although 44.99% of respondents are over 60 years old. While this age and gender distribution may not perfectly mirror the national population structure, it is largely representative of the current demographic reality in rural China. Educational attainment and income levels in the sample are generally low; over 65% have an education level of junior high school or below, and more than 57% report an annual income of less than 30,000 RMB. Regarding the key dependent variable, the overall happiness is positive, with a combined 72.67% of residents identifying as “Happy” or “Very happy.” However, in terms of health metrics, only slightly over half of the residents scored above the general population norm (45) in both physical and mental health dimensions. In terms of accessibility, while physical access appears adequate (79.29% travel < 15 min), the financial burden remains a negative factor, as 26.49% of the sample still faces an out-of-pocket payment-to-income ratio greater than 0.1.

**Table 2:** Descriptive statistics.

Variable	Category	Frequency (*N*)	Percentage (%)
Total sample		589	100.00
Gender	Male	368	62.48
	Female	221	37.52
Age	≤60 years	324	55.01
	>60 years	265	44.99
Marital status	Married	529	89.81
	Unmarried/Single/Other	60	10.19
Occupation	Farmer	289	49.07
	Self-employed	48	8.15
	Employed	70	11.79
	Unemployed/Other	182	30.90
Annual income (RMB)	≤10,000	131	22.24
	10,000–30,000	209	35.48
	30,000–50,000	130	22.07
	50,000–80,000	63	10.70
	80,000–100,000	29	4.92
	100,000–120,000	18	3.06
	>120,000	9	1.53
Education	Primary school and below	189	32.09
	Junior high school	195	33.11
	High school/Vocational	117	19.86
	College/University	84	14.26
	Graduate	4	0.68
Satisfaction with infrastructure	Very dissatisfied	30	5.09
	Dissatisfied	19	3.23
	Neutral	78	13.24
	Satisfied	317	53.82
	Very satisfied	145	24.62
Affordability	>1	16	2.72
	0.5–1	43	7.30
	0.1–0.5	97	16.47
	≤ 0.1	433	73.51
Availability	Always unavailable	133	22.58
	Sometimes unavailable	260	44.14
	Basically satisfied	139	23.60
	Available & sufficient	57	9.68
Geographical accessibility	≤ 15 min	467	79.29
	> 15 min	122	20.71
PCS	≤ 50	289	49.07
	> 50	300	50.93
MCS	≤ 50	253	42.95
	> 50	336	57.05
Happiness	Very happy	110	18.68
	Happy	318	53.99
	Neutral	124	21.05
	Unhappy	25	4.24
	Very unhappy	12	2.04

Table 3 compares the means of selected variables between the two study regions, revealing significant regional heterogeneity. In comparison to rural residents in Yudu, those in Kunshan exhibit significantly better health outcomes and socioeconomic status. Regarding physical health, Kunshan residents’ scores approximate the general population norm, whereas their mental health scores significantly exceed the general population average. In contrast, rural residents in Yudu show a distinct gap below the general population average in both physical and mental health scores. Specifically, Kunshan residents report higher PCS scores (49.39 vs. 45.29) and MCS scores (52.86 vs. 47.23) compared to their Yudu counterparts. Consistent with these health metrics, the self-reported happiness level in Kunshan (Mean = 4.06) is notably higher than in Yudu (Mean = 3.62).

**Table 3:** Selected variables between study regions.

Variable	Mean (Yudu)	Mean (Kunshan)	Std. (Yudu)	Std. (Kunshan)
PCS	45.29	49.39	11.16	8.25
MCS	47.23	52.86	11.82	9.25
Happiness	3.62	4.06	0.85	0.79
Affordability	0.25	0.05	0.41	0.08
Availability	2.54	1.83	0.83	0.83
Geographical accessibility	15.35	11.41	20.29	10.00
Income level	1.93	3.31	1.14	1.41
Education level	2.10	2.28	1.04	1.07
*N* = 589				

Furthermore, substantial disparities are observed in geographical accessibility and affordability. Kunshan residents benefit from shorter travel times to medical facilities (11.41 min) compared to Yudu (15.35 min), reflecting better transportation and healthcare infrastructure in Kunshan. Nevertheless, the findings demonstrate that the spatial layout of rural healthcare services across both regions substantially satisfies the requirements of China’s national strategic initiative to build a “15-minute healthcare circle”. The most striking difference is in the financial burden of medical care between the two places: the mean out-of-pocket payment-to-income ratio in Kunshan is only 0.05, whereas it is five times higher in Yudu. These significant differences indicate that the association between healthcare accessibility and resident happiness may vary across regions due to distinct levels of economic development and social security coverage, a pattern that will be addressed in the subsequent empirical analysis.

### 4.2 Benchmark regression results

Tables 4, 5 present the estimation results of the ordered logit models, examining the multidimensional association between healthcare accessibility and rural residents’ happiness. To ensure the robustness of the estimates and delineate the explanatory power of different correlates, a stepwise regression strategy was employed. Models (1) and (3) include only the core explanatory variables for the Yudu and Kunshan samples, respectively, while Models (2) and (4) further incorporate a comprehensive set of demographic and socioeconomic controls. The inclusion of these control variables notably improved the model fit, increasing the Pseudo *R*^2^ from 0.0628 to 0.1218 for Yudu and from 0.0230 to 0.0486 for Kunshan. The Wald chi-square tests for all specifications rejected the null hypothesis, confirming that the introduction of individual heterogeneity effectively enhances the model’s validity.

**Table 4:** Benchmark results.

Variables	*β*	Std. err.	*z*
Independent variables			
Affordability	−1.392***	0.265	−5.25
Geographic accessibility	−0.010**	0.005	−2.18
Availability	0.202**	0.096	2.11
Control variables			
Gender	0.241	0.18	1.34
Age	0.033***	0.008	4.42
Marital status	−0.077	0.268	−0.29
Education	0.128	0.107	1.2
Income	0.259***	0.076	3.39
Occupation	0.114*	0.063	1.8
Satisfaction	0.353***	0.09	3.94
Health consciousness	0.014***	0.005	2.74
Model fit diagnostics			
Log likelihood	−638.569		
LR *χ*^2^ (11)	121.87		
Pseudo *R*^2^	0.0871		
Observations (N)	589		

***, **, and * denote statistical significance at the 1, 5, and 10% levels, respectively.

**Table 5:** Benchmark results of two regions.

Variables	(1) Yudu (Core)	(2) Yudu (Full)	(3) Kunshan (Core)	(4) Kunshan (Full)
Core variables				
Affordability	−1.161***	−1.034***	−3.807*	−4.805**
	0.237	0.279	1.980	2.060
Geographical accessibility	−0.008	−0.008	−0.006	−0.005
	0.004	0.005	0.014	0.012
Availability	0.652***	0.487***	0.393***	0.351**
	0.159	0.155	0.152	0.164
Control variables				
Satisfaction		0.552***		0.131
		0.129		0.156
Health consciousness		0.023***		0.105
		−0.008		0.007
Model statistics				
Observations	311	311	278	278
Pseudo *R*^2^	0.063	0.122	0.023	0.049
Wald *χ*^2^	46.45	82.18	11.49	37.39

Standard errors are reported in the second column. ***, **, and * denote statistical significance at the 1, 5, and 10% levels, respectively.

#### 4.2.1 Estimation results

The regression results from the pooled full sample (Table 4) initially suggest that all three dimensions of healthcare accessibility—affordability, availability, and geographical accessibility—are significantly associated with rural residents’ happiness. However, a more granular analysis using the split-sample approach (Table 5) reveals substantial regional heterogeneity and structural differences in these patterns of association.

First, affordability emerges as the most robust and dominant negative correlate of happiness across the two places. The coefficient for affordability is significantly negative in both regions, robust to the inclusion of control variables. Notably, the magnitude of this negative association exhibits distinct regional heterogeneity: the absolute value of the coefficient in the developed Kunshan region (*p* = 0.020) is substantially larger than that in the less-developed Yudu region (*p* < 0.001). This disparity suggests that in economically developed areas, despite higher absolute income levels, residents may exhibit a heightened sensitivity to medical economic burdens. This may reflect higher opportunity costs or a stronger sense of relative deprivation when medical expenditures crowd out other consumption.

Second, regarding availability, the density of medical resources significantly and positively correlated with residents’ happiness in both locations (Yudu: *p* < 0.01; Kunshan: *p* < 0.05). This result highlights the universal positive statistical link between supply-side interventions and rural welfare, regardless of the region’s level of economic development.

#### 4.2.2 Geographical accessibility

One critical finding concerns the behavior of Accessibility across different model specifications. While the full sample regression indicates a statistically significant negative association between travel time and happiness (*β* = −0.010, *p* = 0.029), this significance vanishes in the split-sample analysis. In the Yudu sample, travel time shows only a marginally significant negative association in the univariate model (*p* = 0.078) and becomes insignificant after controlling for individual characteristics (*p* = 0.115). Similarly, it remains statistically insignificant throughout the Kunshan models (*p* = 0.680).

This discrepancy between the pooled and split-sample results may reflect aggregation effect and the varying statistical power. The significance observed in the full sample is likely driven by the larger sample size (N = 589), which reduces standard errors and detects a weak, generalized negative trend. However, the lack of robustness in the split samples indicates that, against the backdrop of improved rural infrastructure and transportation, the marginal cost of physical distance has diminished. Geographical accessibility is no longer a primary binding constraint comparable to the economic pressure of affordability or the scarcity of resources. Given its lack of robust explanatory power in the heterogeneous analysis, geographical accessibility will be excluded from the subsequent detailed discussion on regional differences, allowing the analysis to focus on the more salient dimensions of affordability and availability.

Finally, the estimates for control variables further highlight the structural differences in happiness associational patterns. In Yudu, public service satisfaction and health awareness significantly and positively associated with happiness, indicating that happiness in less-developed regions remains heavily dependent on basic public goods and health awareness. Conversely, these variables are insignificant in Kunshan, implying that as regional economies develop and basic services equalize, the determinants of happiness diversify, and traditional indicators of public service evaluation are no longer the sole drivers of happiness.

### 4.3 Comparison of dimensional associations

To further explore the relative importance of the three dimensions of healthcare accessibility, we compared the standardized coefficients from the baseline Ologit model and performed a series of Wald tests to examine the statistical significance of their differences. The regression results indicate that while all three dimensions are significantly associated with the happiness of rural residents, their associative strengths vary substantially.

The Wald test results (Table 6) provide robust statistical evidence for these dimensional disparities. Specifically, the Wald test rejected the null hypothesis that the coefficients of affordability and geographical accessibility are equal (*χ*^2^ = 6.97, *p* = 0.0083), confirming that the negative association of medical costs with happiness is statistically stronger than that of travel time.

**Table 6:** Wald test results.

Variables	*β*	Std. err.	*z*	[95% conf. interval]
Core independent variables				
Affordability	−0.449***	0.073	−6.12	[−0.593, −0.305]
Availability	0.185**	0.088	2.09	[0.012, 0.357]
Geographic accessibility	−0.174**	0.071	−2.44	[−0.313, −0.034]

***, **, and * denote statistical significance at the 1, 5, and 10% levels, respectively. All tests are based on the comparison of standardized coefficients.

In addition, the difference between the coefficients of affordability and availability was highly significant (*χ*^2^ = 27.73, *p* = 0.0000), indicating that reduced financial burdens correspond to a substantially stronger positive association with welfare than merely increasing the density of medical facilities. A significant difference was also observed between geographical accessibility and availability (*χ*^2^ = 10.28, *p* = 0.0013), suggesting that these two spatial dimensions exhibit distinct correlational patterns with residents’ psychological welfare.

### 4.4 Marginal effects analysis

**Figure 1:**
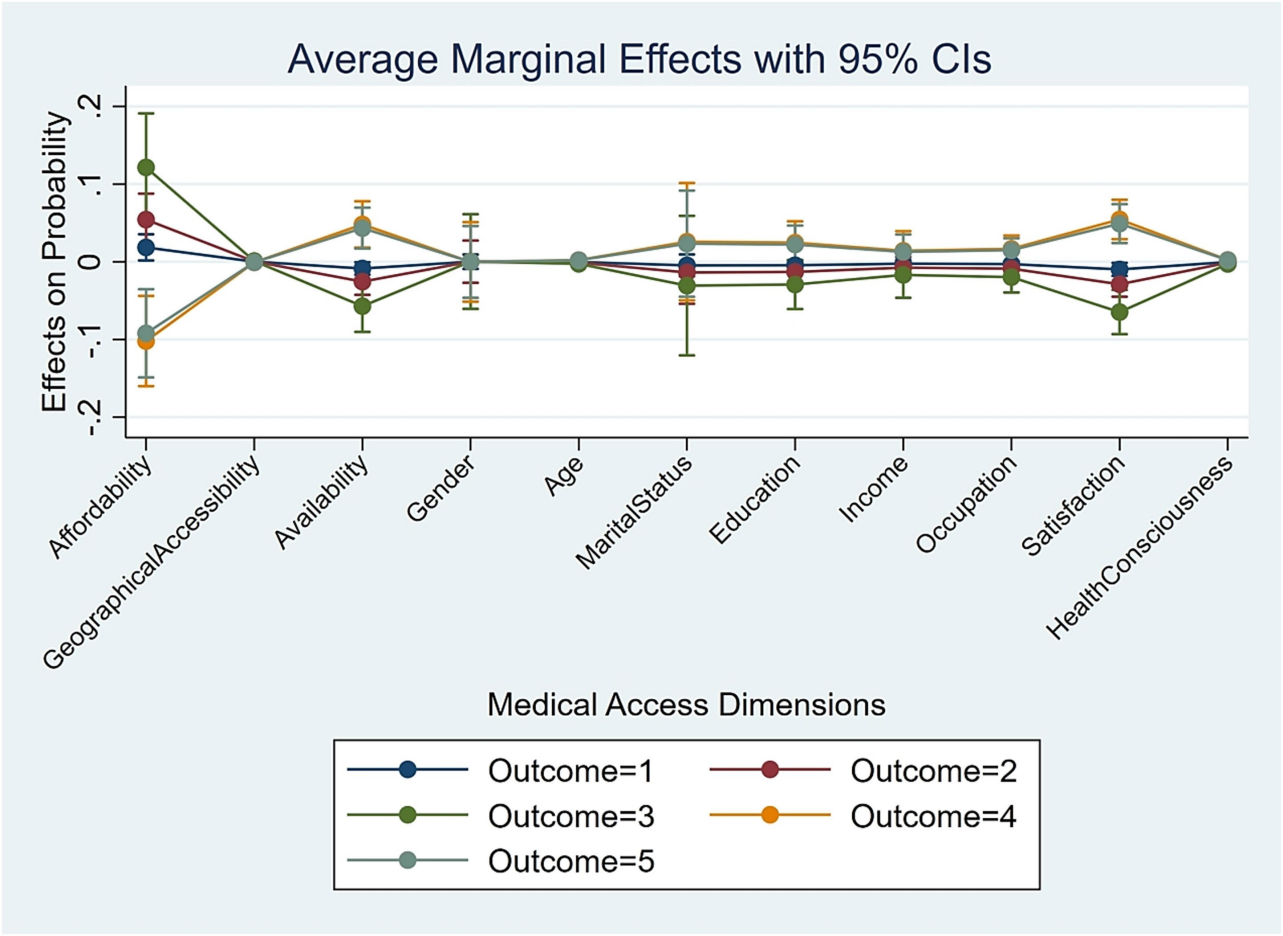
Yudu marginal effect result.

While the ordered logit coefficients establish the direction and significance of associations, and Section 4.3 compares their relative strengths, they do not directly convey the substantive magnitude of these associations on the predicted probabilities of reporting each specific happiness level. To address this limitation and provide a more policy-relevant interpretation of the benchmark results, we computed the Average Marginal Effects (AMEs). The AMEs, derived from the fully controlled models, estimate the change in the probability of being in each happiness category associated with a one-unit higher value in a given predictor, while holding all other variables at their sample means. The results are summarized in Table 7 (Yudu) and Table 8 (Kunshan) and visually presented in Figure 1.

**Table 7:** AMEs on happiness levels: Yudu sample.

Variable/happiness level	Level 1	Level 2	Level 3	Level 4	Level 5
Affordability	0.019**	0.054***	0.121***	−0.102***	−0.092***
Availability	−0.009**	−0.026***	−0.057***	0.048***	0.043***
Geographic accessibility	0.000	0.000	0.001	−0.001	0.000
Satisfaction	−0.010**	−0.029***	−0.065***	0.054***	0.049***
Health consciousness	0.000**	−0.001***	−0.003***	0.002***	0.002***

Values represent AMEs (dy/dx); ***, **, and * denote statistical significance at the 1, 5, and 10% levels, respectively.

**Table 8:** AMEs on happiness levels: Kunshan sample.

Variable/happiness level	Level 1	Level 2	Level 3	Level 4	Level 5
Affordability	0.099*	0.046	0.428***	0.311**	−0.883***
Availability	−0.007*	−0.003	−0.031**	−0.023**	0.065**
Geographic accessibility	0.000	0.000	0.000	0.000	−0.001
Gender (Male = 1)	−0.013*	−0.006	−0.055**	−0.040*	0.114**

Values represent AMEs (dy/dx); ***, **, and * denote statistical significance at the 1, 5, and 10% levels, respectively.

#### 4.4.1 Yudu

The AMEs for Yudu (Table 7, Figure 1) show that a lack of affordability emerges as a critical negative correlate of high happiness. A one-unit increase in the out-of-pocket payment-to-income ratio is associated with a 9.21% decrease in the probability of being “Very happy” (Level 5, *p* < 0.01) and a 10.21% decrease in the probability of being “Happy” (Level 4, p < 0.01). Symmetrically, this sort of financial burden significantly correlated with a higher likelihood of reporting lower happiness levels (Levels 1–3). Conversely, improvements in availability demonstrate a beneficial effect. Greater availability is linked to a 4.80% higher probability of being “Happy” (Level 4, p < 0.01) and a 4.33% higher probability for “Very happy” (Level 5, *p* < 0.01), while correlating with lower probabilities for the lower happiness categories.

Notably, geographical accessibility (travel time) showed no statistically significant marginal probability differences on any happiness outcome (all *p* > 0.10), providing further empirical justification for its exclusion from the core analytical framework as discussed in Section 4.2.2. Among control variables, satisfaction with public services and health consciousness consistently exhibited significant positive marginal associations with the higher happiness levels.

#### 4.4.2 Kunshan

**Figure 2:**
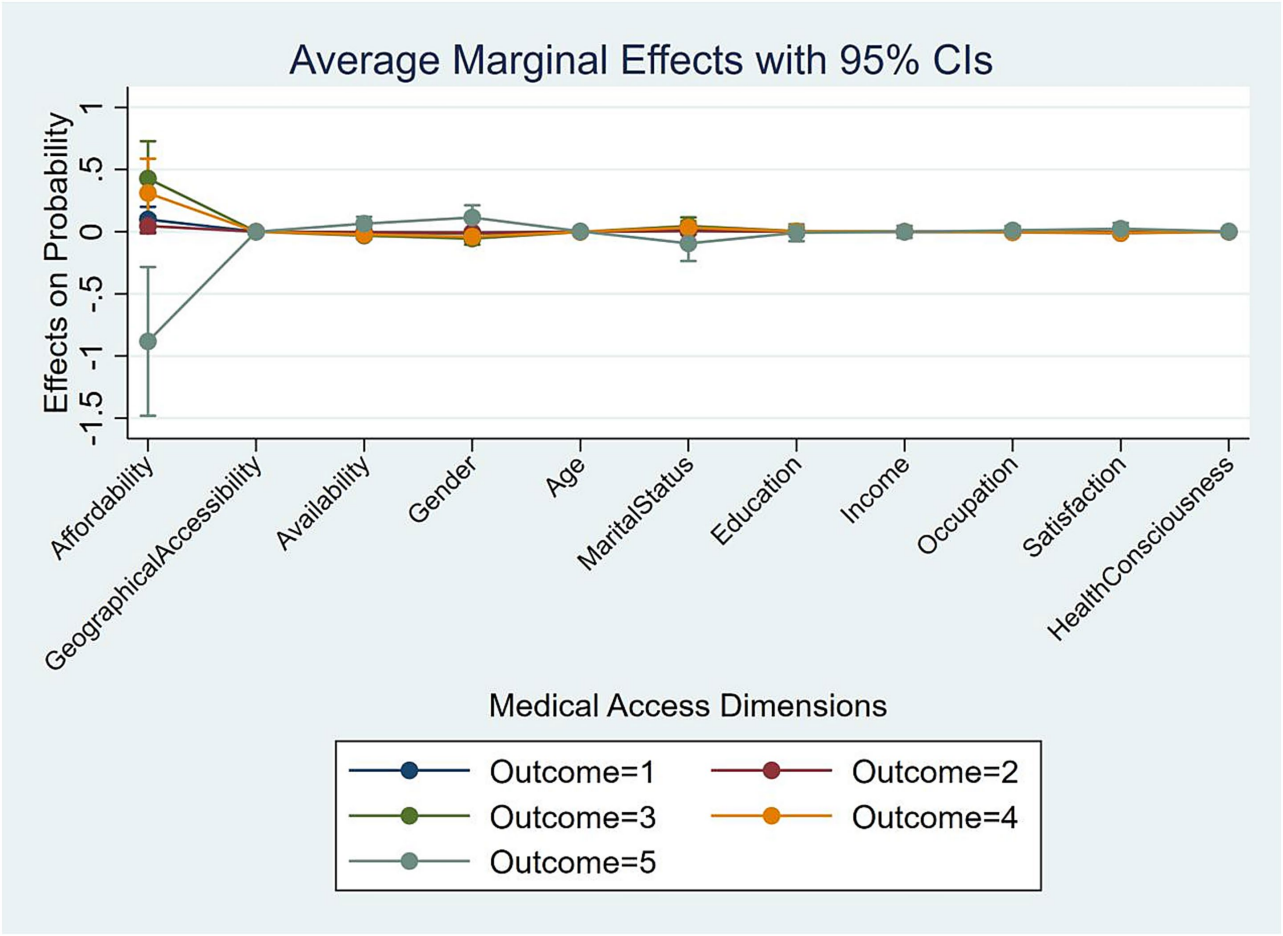
Kunshan marginal effect result.

The marginal effects in Kunshan (Table 8, Figure 2) are not only larger in magnitude but also more complex. The association of affordability with happiness is non-linear. A one-unit higher payment burden is associated with a drastic 88.26 percentage-point lower probability of being “Very happy” (Level 5, *p* < 0.01). This vividly quantifies the heightened sensitivity to medical economic burden in a high-income context. Intriguingly, the same increase is linked to a significant rise in the probability of being in the middle happiness categories (Level 3: +42.76%, p < 0.01; Level 4: +31.07%, *p* < 0.05). This suggests that while high financial pressure is strongly correlated with a catastrophic drop in the likelihood of reporting the highest level of happiness, it is concurrently associated with a greater concentration of the population into a middling state of happiness, highlighting a complex relationship.

The role of availability in Kunshan is muted and inconsistent compared to Yudu. It shows a weak significant positive association with the highest happiness level and small negative associations with Levels 3 and 4.

A salient finding in Kunshan is the significant marginal effect of gender. Being male is associated with an 11.44% percentage-point higher probability of being “Very happy” (Level 5, *p* < 0.05) compared to females, a trend not observed in Yudu.

### 4.5 Propensity score matching

**Figure 3:**
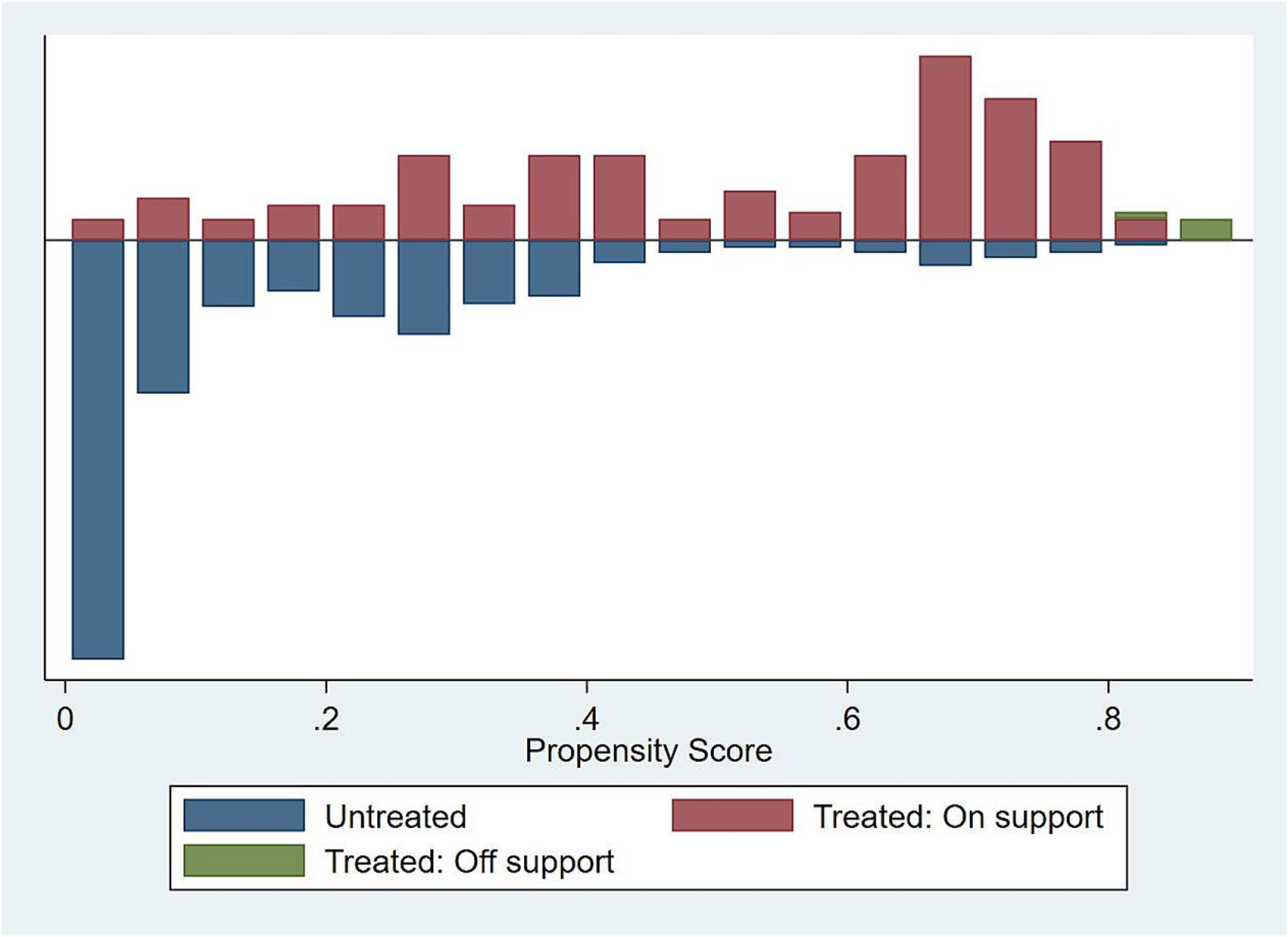
PSM result.

To mitigate the bias in variable selection, this study uses Propensity Score Matching (PSM) established by Rosenbaum and Rubin (46). Specifically, the sample was classified into two groups with high and low affordability, respectively. The treatment group was defined as cases where out-of-pocket medical expenses accounted for more than 10% of the total household income. The result of the test (Figure 3) shows that the probability distributions of propensity scores for the treatment and control groups largely overlap. The vast majority of the observations fell within the common support region, with 585 samples being retained. This indicates an extremely low level of sample loss during the matching process, ensuring that the constructed counterfactual sample possesses high representativeness. The balancing tests confirm the excellent quality of the matching procedure. Specifically, the mean standardized bias was substantially reduced to 9.2%. These diagnostics suggest that PSM effectively reduced bias from observed confounding variables, thereby satisfying the balancing assumption required for quasi-experimental designs.

Based on the valid matched sample, the Average Treatment Effect on the Treated was estimated to examine the core hypothesis. The results show that the treatment effect of a low affordability is associated with a significantly negative difference in happiness, and the T-statistic is significant. This finding implies that, compared to residents with similar characteristics but lower payment ratios, a high payment burden is associated with a tangibly lower level of happiness. This conclusion is highly consistent with the direction and significance of the baseline regression results presented earlier (see Table 9).

**Table 9:** PSM result.

Outcome variable	Sample	Treated	Controls	Difference	*S.E.*	*T*
Happiness	Unmatched	3.44	3.97	−0.54	0.076	−7.03***
	ATT (Matched)	3.45	4.04	−0.59	0.138	−4.30***
Diagnostics						
Matching method	*k*-Nearest neighbor (*k* = 1)					
Observations	On support: 585	Off support: 4				

Standard errors are reported in the second column. ***, **, and * denote statistical significance at the 1, 5, and 10% levels, respectively.

### 4.6 Robustness checks

To verify the robustness of our core findings, this section employs an alternative model specification and excludes extreme samples.

First, to test whether the empirical results are sensitive to ordered logit specification, we use an Ordered Probit model. The estimation results (Table 10) demonstrate remarkable consistency with the previous findings, both in coefficient signs and significance. Affordability maintains a significant positive association with happiness across both regions. The magnitude of the coefficient remains larger in the Kunshan sample. At the same time, the regional heterogeneity regarding medical availability persists across models. This consistency suggests that the observed associative patterns reflect underlying data patterns rather than specific distributional assumptions.

**Table 10:** Robustness check.

Variables	Yudu	Kunshan
Affordability	−0.587***	−2.537**
	0.148	1.085
Geographic accessibility	−0.004	−0.002
	0.003	0.007
Availability	0.248***	0.172*
	0.087	0.093
Controls	Yes	Yes
Observations	311	278
Pseudo *R*^2^	0.119	0.045

Standard errors are reported in the second column. ***, **, and * denote statistical significance at the 1, 5, and 10% levels, respectively. Controls include gender, age, marriage, education, income, occupation, public service satisfaction, and health awareness.

Second, to remove the potential bias introduced by outliers, this study conducted a sensitivity analysis by excluding the subsample falling below the 5th percentile of physical health scores. These regression results (Table 11) corroborate the robustness of our primary conclusions. The negative correlation of the medical payment ratio remains statistically significant and stable in magnitude across both regions. These findings further imply that the core conclusions are not driven by extreme outliers but reflect a generalized fact.

**Table 11:** Robustness check.

Variables	Yudu	Kunshan
Affordability	−0.846*	−4.693**	0.465	2.069
	Geographic accessibility	−0.004	−0.005	0.005	0.012
Availability		0.570***	0.335**	0.163	0.164
	Controls	Yes	Yes
Observations	281	275
R-squared	0.113	0.048

Standard errors are reported in the second column. ***, **, and * denote statistical significance at the 1, 5, and 10% levels, respectively. Controls include gender, age, marriage, education, income, occupation, public service satisfaction, and health awareness.

### 4.7 Regional heterogeneity in mediation pathways

To explore the underlying associative pathways between healthcare accessibility and residents’ happiness, this study adopted the bias-corrected nonparametric percentile Bootstrap method with 2,000 replications. The results indicate that there are significant differences in the mediating patterns across different regions.

As the results show (Table 12), the mediating role of availability in the Yudu area is significantly channeled through the mental health path, with a point estimate of 0.0714 (*p* < 0.001), while the mediating path through physical health was not statistically significant (*p* = 0.361). In contrast, in Kunshan, availability is positively associated with happiness primarily through its correlation with better physical health, with an indirect association magnitude of 0.0366 (*p* = 0.029).

**Table 12:** Bootstrap regression results.

Independent variable	Mediator	Region	*β*	Bootstrap S.E.	*p*	95% conf. interval
Availability	PCS	Yudu	0.013	0.014	0.361	[−0.015, 0.040]
		Kunshan	0.037	0.017	0.029	[0.004, 0.069]
	MCS	Yudu	0.071	0.020	0.000	[0.032, 0.111]
		Kunshan	0.006	0.008	0.417	[−0.009, 0.022]
Affordability	PCS	Yudu	−0.155	0.047	0.001	[−0.247, −0.063]
		Kunshan	−0.328	0.165	0.047	[−0.651, −0.004]
	MCS	Yudu	−0.153	0.049	0.002	[−0.248, −0.057]
		Kunshan	−0.051	0.082	0.532	[−0.211, 0.109]

Regarding the affordability of medical services, the two regions also present distinct patterns. In Yudu, both physical and mental health exhibited mediating roles. The burden of high medical costs significantly is negatively associated with happiness, a correlation linked to both lower physical health (*β* = −0.1548, *p* = 0.001) and mental health (β = −0.1526, *p* = 0.002). However, in Kunshan, the association of affordability was only mediated by physical health (β = −0.3277, *p* = 0.047); the indirect pathway through mental health was not significant (*p* = 0.532).

In conclusion, the pattern of association involving accessibility in the less-developed region is more closely associated with residents’ psychological expectations and dual physical-mental stress, whereas in the developed region, it is predominantly manifested through substantive physical health correlations.

## 5 Discussion

### 5.1 Core findings

The primary objective of this study was to examine the different associative pathways between healthcare accessibility and the happiness of rural residents in China. Our empirical results confirm that both affordability and resource availability are critical correlates of rural happiness, which is consistent with the previous studies (15). However, the most significant theoretical contribution of this study lies in demonstrating that the statistical mediating pathway linking accessibility and happiness is not static but undergoes systematic changes across socioeconomic gradients.

The study first found that although affordability is the most important dimension associated with happiness in both regions, its utility and associative patterns with happiness are different in different regions. In Yudu, the underdeveloped region, the lack of affordability is negatively associated with all levels of rural residents’ happiness. Specifically, the increased burden of healthcare expenditures is associated with a reduced likelihood that residents will enjoy high levels of happiness, while simultaneously showing an increased probability of low levels of happiness. This result aligns with the foundational tier of Maslow’s hierarchy of needs (physiological and safety needs) (47): most of the rural residents in Yudu have a low standard of living, and are dependent on the basic medical services to maintain their physiological health and provide psychological security. In regions where residents’ healthcare needs are primarily for basic survival, increased medical payment ratios fail to ensure psychological security, while rising costs are linked to declining physical health (4) and correspondingly lower happiness (8). Moreover, the crowding-out effect associated with higher medical expenses is further negatively correlated with residents’ happiness (48). This study suggests that in economically disadvantaged areas like Yudu, the growing medical burden is significantly intertwined with constraints on patients’ daily lives—encompassing clothing, food, housing, and transportation—thereby corresponding to a comprehensive lower level of happiness across all dimensions. By investigating mediating factors, we can more clearly elucidate the associative pathways relating to this lower happiness. In resource-constrained environments, a lack of financial affordability is strongly linked to a lack of psychological reassurance regarding easy access to medical care and physical health maintenance (49).

In contrast, the developed Kunshan region presents a counterintuitive phenomenon. Despite a significantly lower objective financial burden and higher income levels, residents there exhibit disproportionate sensitivity to medical costs. The study results indicate that a higher proportion of medical expenses is associated with a significantly disproportionate lower probability of local rural residents achieving the highest level of happiness. Traditional liquidity constraint models fail to fully explain this phenomenon (50); however, it may fit within the loss aversion mechanism of Prospect Theory (45). For instance, in more developed regions, high-level social security and welfare systems are regarded as inherent social components, serving as universal benchmarks for local residents (33). Similarly, Kunshan’s long-standing high welfare standards have become the psychological expectation baseline for rural residents. When medical and related expenditures exceed these expectations, residents may not only perceive them as direct financial burdens but also subjectively experience them as welfare losses (51). Based on loss aversion theory, the psychological negative utility of such losses far outweighs the positive utility of equivalent gains. In developed areas such as Kunshan, where residents have higher quality-of-life expectations, this negative association is particularly pronounced. These findings resonate with the Easterlin Paradox (52), indicating that as socioeconomic development progresses to higher stages, residents’ pursuit of happiness is more likely linked to their psychological expectations (38, 53). Meanwhile, our mediation analysis reveals a possible pattern: this negative association is channeled primarily through physical health pathways. In more developed regions like Kunshan, residents maintain higher baselines for daily health maintenance. When unexpected medical expenditures increase, they directly crowd out continued investments in other physical health capital (such as nutrition or preventive care, aligning with Grossman’s model). Therefore, in developed areas, this negative association is particularly pronounced not because of existential psychological panic, but due to tangible constraints on maintaining their expected high standards of physical happiness.

The results also demonstrate that the value of healthcare resource accessibility varies across contexts, evolving from basic survival support to a functionally oriented focus on efficiency enhancement. In underdeveloped regions like Yudu, increased medical resources are significantly associated with higher residents’ happiness, with the mediation analysis showing that this associative pattern is channeled entirely through mental health pathways. This aligns with the option value concept in public economics (54), indicating that in areas with limited medical resources, where healthcare resources inherently function as a safety net (55), accessibility primarily serves as a psychological safeguard for local residents (21). For Yudu residents, improved access to medical resources is strongly linked to lower psychological health anxieties, rather than merely correlating with actual healthcare-seeking behaviors (56, 57), while in developed regions such as Kunshan, basic healthcare accessibility has been widely achieved. At this stage, since the supply of basic medical resources approaches saturation, the associative strength between mental health and happiness is notably weaker, with happiness showing only a limited correlation with physical health status. The value focus of accessibility has undergone a fundamental shift, with residents now perceiving it as a productive tool for safeguarding and enhancing health capital. Its significance is linked not to the elimination of anxiety, but to the support of more efficient and high-quality health interventions to fulfill specific instrumental objectives (28).

Crucially, the interpretation of these divergent mediation pathways must be contextualized within the demographic realities of the analytical sample. The dataset exhibits a pronounced aging trend (44.99% aged >60) and a male majority (62.48%), effectively mirroring the demographic structure of contemporary ‘hollowed-out’ rural China. These structural characteristics provide a plausible lens for understanding the dominant pathways observed. In resource-constrained regions like Yudu, the significant mediation of both affordability and availability through the mental health pathway aligns conceptually with the heightened physiological and psychological vulnerabilities of an aging demographic, for whom healthcare accessibility primarily functions as an essential buffer against anticipatory anxiety. Conversely, in developed settings like Kunshan, the mediation exclusively through physical health, reflects a distinct shift towards instrumental utility. While the current empirical design lacks the interaction terms necessary to isolate specific demographic drivers, one cautious interpretation is that in affluent regions where basic medical survival needs are saturated, healthcare is utilized more instrumentally to maintain functional physical capacity. Given the male-dominated nature of the sample, this physical-health-oriented mechanism might reflect the priorities of rural residents—striving to sustain their labor participation and socioeconomic stability. However, this remains a speculative hypothesis. Future longitudinal studies incorporating formal gender and age interaction models are required to explicitly verify how demographic intersections shape these healthcare-happiness pathways.

A finding with significant implications is that geographical accessibility did not demonstrate robust statistical significance in either regional sample of this study. This stands in sharp contrast to previous research (19). This discrepancy precisely highlights the practical significance of this study: in present-day China, characterized by substantial infrastructure upgrades, the relative statistical importance of geographical proximity to happiness appears minimal. However, this finding should be interpreted with caution. The reliance on self-reported travel time may introduce recall bias and fail to fully capture perceived latent barriers, such as complex terrain, traffic conditions, or transportation costs. It remains plausible that objective spatial assessments, such as Geographic Information System (GIS) measurements, could uncover different correlational patterns. Nevertheless, within the current methodological scope, widespread improvements in transportation infrastructure appear to have diminished the salience of self-reported distance, allowing it to be superseded as a primary correlate by affordability and availability (58). Meanwhile, this result is consistent with China’s recent policy of establishing a comprehensive healthcare service network. Therefore, this finding supports a strategic shift from pursuing mere geographical universal coverage to striving for quality access.

Based on the ratiocination of a regional gradient in the associative patterns between healthcare accessibility and happiness, this study suggests that public health governance should transition from a one-size-fits-all expansion toward stage-specific, precision-driven intervention strategies. Given that geographic distance no longer emerges as a significant constraint correlated with happiness due to universal rural infrastructure connectivity, the focus of future public health investment should formally shift from spatial coverage to service quality and financial risk management.

In underdeveloped regions where healthcare functions as a primary safety net, policy interventions should prioritize ensuring consistent and reliable access to care. Since the happiness of rural residents in these areas is strongly associated with the mere presence of resources, health authorities should implement a functional visibility mandate for primary clinics. This involves ensuring a consistent supply of essential medicines through digitized inventory monitoring and maintaining stable staffing of general practitioners. Such measures maximize the precautionary value of infrastructure, mitigating anticipatory anxiety regarding health shocks. Simultaneously, to address the crowding-out effect of medical costs on basic survival, authorities should expand catastrophic health expenditure protections, specifically by implementing immediate point-of-service reimbursement for low-income households.

In contrast, in developed rural areas where basic access is saturated, the policy orientation should shift toward instrumental utility and expenditure predictability. As marginal gains from physical expansion diminish, health systems should transition toward a performance framework based on clinical outcomes. This requires incentivizing higher-tier medical resources to decentralize into rural communities, focusing on chronic disease management and rehabilitation to meet higher demand for health capital maintenance. Furthermore, to counter the loss aversion associated with out-of-pocket volatility, the government should promote supplementary health insurance schemes and encourage universal participation. This would convert sporadic, high-cost medical events into predictable, fixed annual premiums, serving as an effective buffer against the sharply lower happiness associated with financial shocks and social reference imbalance.

### 5.2 Limitations and future directions

This study has several limitations that warrant consideration. First, the reliance on cross-sectional survey data inherently precludes the establishment of causal relationships and leaves the pathways susceptible to reverse causality. For instance, it is plausible that individuals with higher baseline well-being perceive their health status more positively or report their financial burdens as less severe. Additionally, the use of self-reported data introduces the potential for common method bias, given that both the independent and dependent variables were assessed using the same survey instrument. While this study identifies significant statistical associations, future research employing longitudinal designs and objective healthcare metrics—such as GIS mapping and personnel allocation data—is necessary to address reverse causality, capture objective spatial dynamics, and enhance overall measurement validity.

Second, while the two-county comparative framework and the sample’s demographic profile offer a representative context for contemporary ‘hollowed-out’ rural China, from a universal perspective, the demographic characteristics and gender composition of the sample in further research also require additional refinement. Meanwhile, the empirical design precludes formal testing of spatial and demographic heterogeneity. Given China’s vast geographical diversity, the identified mediation pathways may exhibit regional variations beyond the scope of these selected sites. Furthermore, the absence of statistical interaction terms (e.g., Age × Affordability) limits the ability to isolate specific demographic drivers, rendering the proposed mechanisms exploratory. Future research should employ expansive multi-regional sampling and formal interaction modeling to substantiate these healthcare-happiness pathways across broader geographic and demographic contexts.

## 6 Conclusion

This study demonstrates that healthcare accessibility is associated with variations in rural happiness through dimensional differentiation and economic gradient evolution. While geographic distance is no longer significantly associated with lower happiness, affordability remains the primary correlate of reduced happiness in both regions. Importantly, the mediation analysis reveals significant regional heterogeneity in these associative pathways. In underdeveloped areas, healthcare access functions primarily as a psychological safety net, with its positive association with happiness channeled through mental health pathways by mitigating anxiety. In contrast, in developed regions, the mechanism shifts toward an instrumental orientation, where accessibility is linked to happiness predominantly through the maintenance of physical health capital. This multidimensional framework provides a nuanced understanding of the evolving internal happiness disparities in transitional rural China.

## Data Availability

The raw data supporting the conclusions of this article will be made available by the authors, without undue reservation.
